# An Exploratory Analysis of the Roles of Nurses on a Pediatric Rehabilitation Unit in South Korea Perceived by Pediatric Rehabilitation Professionals

**DOI:** 10.3390/healthcare12020177

**Published:** 2024-01-11

**Authors:** Hanna Lee, Da-Jung Kim, Jeong-Won Han

**Affiliations:** 1 Department of Nursing, Gangneung-Wonju National University, Wonju-si 26403, Republic of Korea; hannalee@gwnu.ac.kr; 2 Department of Nursing, Cheju Halla University, Jeju-si 63092, Republic of Korea; djk@chu.ac.kr; 3 College of Nursing Science, Kyung Hee University, Seoul 02447, Republic of Korea

**Keywords:** nurse, pediatric nursing, rehabilitation, role, South Korea

## Abstract

Rehabilitation addresses not only children’s disabilities but also their physical, psychological, social, and cultural impairments. Hence, pediatric rehabilitation adopts a multidisciplinary approach; it encompasses the vital role of not only physicians and rehabilitation therapists, but also of nurses. This study conducts a content analysis of the experiences of healthcare professionals specializing in pediatric rehabilitation to explore the roles nurses working on pediatric rehabilitation units are expected to perform. After analyzing the interviews with 12 experts in pediatric rehabilitation, the roles of pediatric rehabilitation nurses were broadly categorized into five areas (caregivers, team members, counselors, researchers, and educators) with eight sub-groups and 24 specific roles. This study is significant because it provides profound insights into the roles of pediatric rehabilitation nurses in Korea. These insights can serve as foundational data for formulating policies for healthcare personnel in pediatric rehabilitation, and provide evidence for establishing a much-needed system for certified rehabilitation nurses in Korea.

## 1. Introduction

As of 2021, the number of registered individuals with developmental disabilities in South Korea was 252,000, representing an increase of 18,000 from 2018 [1]. Out of these, approximately 300,000 were children [2]. According to the Ministry of Health and Welfare National Survey on Persons with Disabilities, nearly 100,000 children with disabilities are on the waitlist for rehabilitation in Korea [2]. These data demonstrate the growing societal demand for pediatric rehabilitation services in response to the rising number of children with disabilities. Consequently, pediatric rehabilitation has expanded in clinical settings. Pediatric rehabilitation services cater to children under the age of 18 with various disabilities, including visual, auditory, speech, intellectual, autistic, and neurological disorders. Their primary objective is to help develop and enhance cognitive, communication, adaptive behavior, sensory, and motor skills [3]. They also focus on minimizing impairments and facilitating age-appropriate development in children who either have disabilities or are at risk of developing them due to illnesses or accidents in their early years, when growth and development are ongoing [4]. Healthcare professionals specializing in pediatric rehabilitation play a multifaceted role. They address not only the physical health issues of children, but also assume other responsibilities to support rehabilitation processes [5].

However, the delivery system for rehabilitative medicine in Korea lacks a clear definition. Rehabilitation services are provided in various healthcare facilities, ranging from tertiary hospitals, which primarily offer acute care, to long-term care hospitals, which help maintain functionality during chronic phases. Pediatric rehabilitation is provided either on a single unit or within a designated center in tertiary hospitals, or select primary and secondary care facilities [6]. Furthermore, in Korea, rehabilitation nursing meant for adults and children is not distinctly differentiated, and is broadly categorized into areas such as “neurogenic bladder management”, “neurogenic bowel management”, “dysphagia management”, and “respiratory rehabilitation nursing”. However, considering that children and adolescents differ from adults in physical, psychological, and social aspects, it is crucial to specify the scope of rehabilitation nursing and the role of nurses in pediatric rehabilitation based on the growth phase [7]. Especially in Korean healthcare institutions, where nurses often rotate through different units instead of working on only one unit, there is a need for educational materials and training that provide clear guidance on the tasks and roles nurses should undertake when assigned to a pediatric rehabilitation unit.

In contrast, in the United States, the Association of Rehabilitation Nurses outlines the roles and responsibilities of pediatric rehabilitation nurses, which encompass advocacy, care coordination, leadership and consultation, direct care provision, health education and promotion, teamwork, research, and professional practice, education, and evaluation [8]. In the United States, pediatric rehabilitation nurses are certified professionals with relevant knowledge and skills. Moreover, various education and certification programs are available in the pediatric nursing field, such as Certified Rehabilitation Registered Nursing, Registered Nurse Certification in Developmental Disabilities, Certified Pediatric Nursing, and Clinical Nurse Specialist and Certified Pediatric Nurse Practitioner qualifications [9].

Rehabilitation addresses not only children’s disabilities but also their physical, psychological, social, and cultural impairments. Hence, pediatric rehabilitation adopts a multidisciplinary approach; it encompasses the vital role of not only physicians and rehabilitation therapists, but also nurses. It is crucial to understand the roles nurses are expected to perform within this multidisciplinary team [10]. Given these considerations, it is essential to identify the specific roles pediatric rehabilitation nurses are expected to perform in Korea, and determine the direction of pediatric rehabilitation nursing education and training in both clinical practice and school curricula. The first step in this endeavor is to systematically explore and understand how healthcare professionals currently working in the pediatric rehabilitation field perceive the roles of nurses working on pediatric rehabilitation units. This study aims to explore these roles from the perspectives of various healthcare professionals, considering that clinical nurses may have been assigned to work on pediatric rehabilitation units at some point in their careers due to the rotational nature of nursing assignments in all units in Korean hospitals.

## 2. Methods

### 2.1. Study Design

This study conducts a content analysis of the experiences of healthcare professionals specializing in pediatric rehabilitation, using qualitative research methods to explore the roles nurses working on pediatric rehabilitation units are expected to perform.

### 2.2. Study Participants

We arbitrarily selected one rehabilitation hospital in S City and one tertiary hospital in G Province. Individuals who voluntarily provided informed consent were selected. A total of 12 participants were enrolled; they comprised 8 nurses, 2 rehabilitation medicine specialists, and 2 pediatric rehabilitation therapists working in the rehabilitation hospital or on the rehabilitation medicine unit in the tertiary hospital. The inclusion criteria were as follows: (1) professionals specializing in rehabilitation therapy and nursing (physicians, nurses, physical therapists, or occupational therapists) who provided informed consent to participate in the study; (2) physicians with at least 10 years of experience of working in a rehabilitation hospital or rehabilitation medicine in a tertiary hospital; and (3) nurses or rehabilitation therapists with at least five years of experience of working with pediatric patients undergoing rehabilitation.

### 2.3. Data Collection

#### 2.3.1. Focus Group Interviews

Data were collected using focus group interviews. We formed 3 focus groups consisting of 3–5 participants with varying occupations, and the interviews began with a greeting and introduction from the researcher. The interviewer used semi-structured and open-ended questions and avoided intentionally guiding participants’ responses to desired answers. The key interview questions were “What do you think pediatric rehabilitation nursing is?”; “What is expected from pediatric rehabilitation nurses?”; and “What do you think is the job description for pediatric rehabilitation nurses?”. Participants were encouraged to freely discuss the topic, and a list of additional follow-up questions was prepared to facilitate the interview as needed. The researchers listened to the recorded interviews repeatedly and transcribed them verbatim. The data collection period was conducted for three groups on 5 June, 7 June and 10 June 2023, respectively. Before obtaining written consent, the participants were informed about the study’s purpose and process and informed that the interviews would be audio- and video-recorded. An additional question-and-answer session was held to inform participants that participation was completely voluntary and that they could withdraw from the study at any time. The focus group interviews were conducted for each of the three groups at an office near the hospital, at a quiet cafe without external disturbances, and in online video interviews. The focus group interview took approximately 60 to 90 min.

#### 2.3.2. In-Depth Interviews

In-depth interviews were additionally conducted with participants who attended the focus group. They were conducted to confirm individual opinions that were not found in the focus group. The purpose of these interviews was to validate individual opinions that might not have been fully expressed or explored within the group setting. While focus groups provided valuable insights through group dynamics, the in-depth interviews allowed us to delve deeper into each participant’s perspective, capturing nuances that might not have surfaced in the collective discussion. In-depth interviews were conducted by the researchers, and data were collected until saturation was reached, that is, until no new information emerged. We collected data between 15 June 2023, and 30 June 2023. There were a total of 5 participants. Interviews were mainly conducted through mobile video calls or online video conferences. The number of interviews was once per person, and the interview time lasted from a minimum of 25 min to a maximum of 45 min. The interviews concluded when participants stated that they had nothing more to add. The data collected from these interviews were interview transcripts and debriefing notes the researchers prepared immediately after the interviews.

### 2.4. Data Analysis

The collected data were analyzed using qualitative content analysis. Qualitative content analysis is an inductive analytical approach that does not adhere to specific theoretical or philosophical frameworks, such as grounded theory or phenomenology. Instead, it focuses primarily on the inherent meaning within the data, guided by the research question [11]. This methodology efficiently categorizes extensive datasets based on the research objective, extracting categories that encapsulate similar meanings. Qualitative content analysis is utilized to acquire knowledge and an understanding of the phenomenon of interest, particularly when there is a dearth of pertinent literature. This analytical method is designed to inductively elucidate aspects such as participants’ attitudes, values, behaviors, interests, and motivations and factors impacting their actions from their own perspectives. Thus, this approach aligns well with the objectives of our study, which seeks to investigate the role of nurses in pediatric rehabilitation by drawing from the experiences of various healthcare professionals specializing in pediatric rehabilitation. We transcribed participants’ statements verbatim and analyzed data solely based on the transcripts, with a concerted effort to minimize the influence of theory, preconceived notions, and biases. The overarching aim was to attain a comprehensive understanding of the phenomenon of interest.

### 2.5. Reliability

We systematically analyzed and evaluated this qualitative study using the three-step approach outlined in the Consolidated Criteria for Reporting Qualitative Research, as presented in the Equator network library [12]. We have completed courses and education on qualitative research, attended various seminars on qualitative research, conducted qualitative research in the past, have experience in pediatric rehabilitation nursing, or have conducted research on pediatric care. We practiced active listening during the interviews, ensuring that we did not guide participants’ responses in a certain direction. We also shared our notes during data collection and analysis for discussion, ensuring that researchers’ field experiences and pre-existing knowledge did not serve as prejudices during the study process.

### 2.6. Rigor

We ensured the trustworthiness of this qualitative research based on the criteria outlined by Lincoln and Guba [13], which include credibility, transferability, auditability, and confirmability. To enhance credibility, extensive amounts of data were collected by well-seasoned nurses qualified for the purpose of this study by leveraging their communication skills. Additionally, we regularly self-reflected on our biases and documented these reflections to create an atmosphere where participants could freely share their experiences without undue influence. We also upheld this attitude during the data analysis phase. To minimize inaccuracies and incompleteness in the data, a two-step interview method was employed. After the initial interview, the interview transcript was reviewed, and follow-up questions were posed during a second interview. The research findings derived from these follow-up interviews were then compiled into a list and re-verified by the participants. Transferability refers to how well the study findings can be applied to other situations and contexts. In this study, we presented the general characteristics of the participants, the study procedures, and the results in detail to allow readers to assess and explore the applicability of the research findings to their own contexts. Auditability is the criterion for assessing the consistency of the research process, including research time, duration, data collection, analysis methods, and the appropriateness of the researcher’s involvement. We addressed this by making field notes and analysis notes and reviewing and cross-validating them. Confirmability is a concept for safeguarding the neutrality of the research by minimizing fundamental values, biases, and prejudices held by the researchers, and it is interconnected with credibility, transferability, and auditability. We aimed to achieve this goal through consistent self-reflection, meticulous note-taking, and close communication with team members throughout the research process. Additionally, we enhanced confirmability by allowing the participants to verify the results.

## 3. Results

### 3.1. Participants’ Characteristics

The mean age of the 12 participants was 37.17 years. Ten participants were in their 30s, one in their 20s, and one in their 40s. All participants were female. Eight were nurses, two were rehabilitation medicine specialists, one was a physical therapist, and one was an occupational therapist. On average, participants’ total clinical career was 14.2 years, and their career in rehabilitation was 11.6 years (Table 1).

### 3.2. Exploratory Analysis of the Roles of Pediatric Rehabilitation Nurses

**Figure 1:**
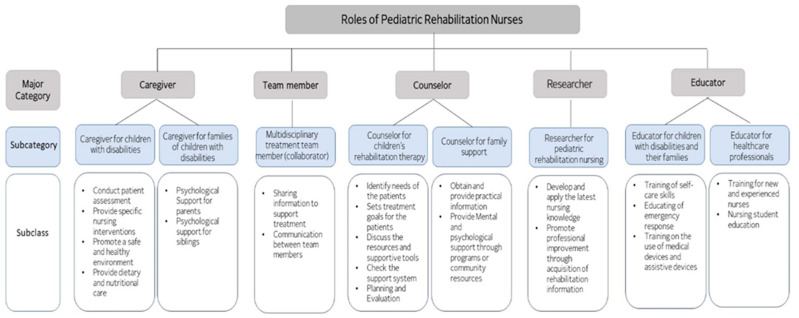
Exploratory analysis of the roles of pediatric rehabilitation nurses.

After analyzing the interviews with 12 experts in pediatric rehabilitation, the roles of pediatric rehabilitation nurses were broadly categorized into five areas with eight sub-groups and 24 specific roles (Figure 1).

#### 3.2.1. Caregiver

The participants broadly categorized pediatric rehabilitation nurses’ role as caregiver into caregivers for children with disabilities and caregivers for their families.

##### Caregivers for Children with Disabilities

The role of caregiver for children with disabilities included “patient assessment”, in which the nurse identified the differences between children without disabilities and those with disabilities, and determined the severity of the disability. As caregivers, the nurses also “performed nursing interventions”, which included changing the patient’s position according to their need, administering medication, maintaining range of motion, and performing skin care. The nurses also “fostered an environment of physical safety and well-being”, provided “diet and nutritional care”, and monitored and recorded patient status. However, relevant education or educational materials were lacking, which posed challenges for nurses who were new to pediatric rehabilitation.

*“Yes, pediatric rehabilitation is a specialized unit, but I think it’s important to know the basics of the practice. Pediatric rehabilitation patients take several medications, so issues like medication errors should never happen. However, there are many patients who are ordered special injections, like botox or phenol, and there are definitely skills that are specific to pediatric rehabilitation, but it’s hard because there are no manuals or books to learn these from”. (Participant 12)*

*“(Omitted) I think you need to clearly know the difference from normally developing children, and when providing care to pediatric rehabilitation patients, you need to be able to differentiate them”. (Participant 9)*

##### Caregiver for Families of Children with Disabilities

The participants expressed that pediatric rehabilitation nurses play the role of a “caregiver for the families of children with disabilities”. Disability and rehabilitation cannot be addressed in the short term. Rehabilitation requires long-term dedication and commitment from the family, but parents often become mentally exhausted during this process. Moreover, the patient’s siblings may feel neglected and fail to receive proper care during their growing years. Thus, nurses must provide psychological support to the families of children with disabilities and guide them toward support programs. Early intervention for psychological issues is crucial to promote resilience, as it may positively impact the child’s rehabilitation process. The nurses also mentioned that programs for siblings can help strengthen family bonding while tending to the sibling’s needs. To be able to perform the role of a caregiver, building trust within the family is the most important, which requires communication and interpersonal competencies.

*“I met a boy who accompanied his little brother undergoing rehabilitation. The boy was an elementary school kid, and he looked intimidated and sad. I had a chance to talk to him, and he said that he doesn’t get enough attention from his parents because his parents focus only on his little brother. I felt really sorry for him. I think providing care that includes the siblings of pediatric patients is also within the scope of our practice”. (Participant 4)*

*“Since the rehabilitation process is long, the patient’s mom, who was the primary caregiver, seemed to be burnt out. I felt sad watching the patient’s mom having no motivation and constantly crying. (omitted) I provided emotional support and continued to pay attention to her, and she got a little better… I think nurses play a huge role in this aspect as well”. (Participant 7)*

#### 3.2.2. Team Members

##### Multidisciplinary Treatment Team Members (Collaborators)

The participants mentioned that “multidisciplinary team member” is a role that pediatric rehabilitation nurses must perform. They emphasized that clearly recognizing their role as a team member and providing necessary therapeutic support is essential. They stated that nurses are healthcare professionals who spend the most time with the child with disabilities, and they optimize the patient’s treatment by collaborating with physicians, physical therapists, occupational therapists, speech therapists, and other healthcare professionals. In particular, as nurses come across various types of information, such as patients’ and families’ needs and responses to treatment, they are expected to share such information with the team and facilitate communication between the patient and other healthcare providers.

*“They say that rehabilitation is a multidisciplinary area. I see that hands-on. It’s important for many experts. For instance, physicians, nurses, pharmacists, physical therapists, occupational therapists, speech therapists, social workers, and nutritionists need to share their opinions and collaborate for one patient”. (Participant 1)*

*“(Omitted) There are many times when a nurse stands next to the caregiver and speaks for the patient. Sometimes, it’s easier to understand than when one has to talk to the caregiver directly because (nurses) summarize the essential things (laughs)”. (Participant 12)*

*“Out of all healthcare professionals, nurses spend the most time with the patient and caregiver. Then, when they build a rapport with the caregivers of the pediatric rehabilitation patient, we sometimes depend on the nurses. When we ask, what do we do in this case? The nurses help us to make objective judgments”. (Participant 2)*

*“However, I wish that the nurses were well aware of why rehabilitation therapy is needed. Many of them do not know”. (Participant 9)*

#### 3.2.3. Counselors

Another role that the participants described was that of a counselor who ensures continuity of care. They mentioned that nurses serve as counselors for “children’s rehabilitation therapy” and “family support”.

##### Counselors for Children’s Rehabilitation Therapy

Serving as a “counselor for children’s rehabilitation therapy” involves setting the children’s and family’s expectations for rehabilitation in the healthcare facility and the anticipated effects. The nurse first identifies their needs and sets treatment goals for the patient. Then, they contemplate treatment techniques and methods based on the set goals, and discuss the resources and supportive tools required for the treatment with the multidisciplinary team. The participants emphasized the importance of nurses’ role as a counselor in implementing and evaluating rehabilitation therapy plans.

*“It’s sad, but pediatric rehabilitation in most cases continues from childhood to adulthood. It’s important to discuss what the family wants from the beginning and consider those needs to take a long-term approach that will continue into adulthood. What we need here is that the nurse must be also informed about adult rehabilitation to provide relevant information to the family as needed”. (Participant 6)*

##### Counselors for Family Support

The role of a “counselor for family support” involves helping the child maintain their social and daily life after they have completed treatment at the healthcare facility. Pediatric rehabilitation nurses must obtain and provide practical information, such as assistive devices needed for rehabilitation, walking aids, rehabilitation products, educational materials, emergency healthcare facilities, bedside services, and information about social support systems for the family. The participants also stated that nurses must provide mental and psychological support to the family, and connect them with programs or community resources that can help them resume their social lives.

*“(omitted) You know, pediatric rehabilitation is mostly available in the Seoul metropolitan area. There are many patients who complete treatment to a certain extent here and then return to their hometowns to continue receiving rehabilitation therapy. When they first go to a hospital in their local area, we get a lot of calls about drugs and treatment. Then, we explain it to them”. (Participant 4)*

#### 3.2.4. Researchers

##### Researchers for Pediatric Rehabilitation Nursing

The participants emphasized that pediatric rehabilitation nurses must also serve as a “researcher in pediatric rehabilitation nursing”. The nurses strive to advance their competence and skills by studying pediatric rehabilitation, which includes reviewing relevant literature, attending seminars such as academic conferences, and seeking further education. However, they noted that papers on pediatric rehabilitation are relatively scarce in Korea, which makes it difficult for them to utilize educational materials and evidence from clinical practice.

*“When I review existing studies, there aren’t many studies on pediatric rehabilitation patients. Is it because the study population is so specific? I think I need to be aware of the latest findings… That’s one downside”. (Participant 5)*

*“I attended a conference at the Korean Society of Rehabilitation Nursing, but it was unfortunate that everything was about adult rehabilitation and there was nothing about pediatric rehabilitation. Is it because they don’t think it has a great impact?…”. (Participant 4)*

#### 3.2.5. Educators

The participants highlighted that pediatric rehabilitation nurses must perform the role of an “educator”, both an “educator for children with disabilities and their families” and an “educator for healthcare professionals”.

##### Educators for Children with Disabilities and Their Families

The role of a pediatric rehabilitation nurse as an “educator for children with disabilities and their families” involves educating the children and their families about self-care, providing educational materials for reference as needed, and educating them about using and managing medical and assistive devices in daily activities. Furthermore, the participants stated that the nurse plays an essential role in educating patients and their families about seeking help in emergencies and dealing with such situations. However, in reality, nurses face challenges in performing this role due to a lack of educational media and materials.

*“You know, providing care to children with disabilities is a long-term process that spans from childhood to adulthood. So, I believe nurses educate the parents about various things, from the difference between children with and without disabilities, fostering the child’s potential despite the disability, dealing with emergencies, to the scope of daily life activities”. (Participant 3)*

##### Educators for Healthcare Professionals

The participants also discussed the role of a pediatric rehabilitation nurse as an “educator for healthcare professionals”. They emphasized the importance of imparting the latest knowledge and skills in pediatric rehabilitation nursing to both new and experienced nurses, along with educating healthcare professionals about effective communication methods for interacting with children with disabilities, their families, and the multidisciplinary team. However, owing to a decline in the fertility rate, the reduction in the number of pediatric units in healthcare facilities has reduced the percentage of nurses dealing with pediatric patients. Consequently, the participants felt that nursing professionals with expertise in this field are limited. They also stated that the nurses face difficulties due to a lack of educational media, data, and systems needed to provide this education.

*“Not many nurses have had experience in pediatric rehabilitation, so it is difficult to train new nurses. This makes it harder for new nurses. There is a growing need for pediatric rehabilitation, but there is only a limited number of nurses who have the competence. I wish that schools and hospitals educate all nurses”. (Participant 1)*

*“(omitted) The biggest problem is that there is no manual for education. It would be great if it’s equal across all hospitals, but I wish there was a guideline for reference, and I think it’s important to share the knowledge with nurses and physicians on other units based on that”. (Participant 6)*

## 4. Discussion

In this study, we conducted a content analysis to explore the roles that nurses working on pediatric rehabilitation units are expected to perform based on the experiences of healthcare professionals specializing in pediatric rehabilitation. The key findings are discussed as follows. First, nurses are expected to perform the role of a caregiver for children with disabilities and also their families. This finding aligns with the “care provider” role that the ARN suggests for pediatric rehabilitation nurses [8]. It emphasizes that pediatric rehabilitation nurses must provide care to children with disabilities. They must utilize their knowledge and skills in rehabilitation nursing to help these children reach their maximum potential and become active contributors to their families and society. Furthermore, they must consider various aspects, such as physical, emotional, social, cultural, educational, developmental, and spiritual factors, to promote the child’s overall health. Given that the client population comprises growing children, the practice of pediatric rehabilitation nursing should be grounded in developmental theories, and nurses should possess in-depth knowledge about normal child development and relevant assessment methods [14,15]. Therefore, it is essential for academic associations and hospitals to build educational systems that allow nurses to acquire comprehensive knowledge about child health and enhance their expertise. Additionally, these education systems should be accessible to not only nurses, but also other healthcare professionals specializing in pediatric rehabilitation and the families of children with disabilities.

Second, our findings showed that pediatric rehabilitation nurses must serve as integral members of a multidisciplinary team. This role mirrors the “team member” role the ARN suggests [8]. It emphasizes that pediatric rehabilitation nurses should collaborate closely with other professionals, patients, and families to develop, implement, and evaluate interdisciplinary treatment plans. This approach can lead to a service delivery model that most effectively meets the needs of the children and their families. Moreover, a study on rehabilitation nurses reported that nurses working in rehabilitation settings collaborate with many other healthcare professionals, including physicians, physical therapists, and speech therapists. However, this can lead to role conflicts, since nurses prioritize the needs of different stakeholders, such as physicians, other healthcare professionals, and patients, and role conflicts can be a significant source of stress [16,17]. To help nurses perform the role of a team member in a multidisciplinary team, they must be educated about aspects such as organizational communication and conflict resolution.

Third, we found that pediatric rehabilitation nurses must serve as counselors to ensure the continuity of treatment. This role aligns with the “leader and consultant” role proposed by the ARN [8]. Healthcare professionals who participated in this study talked about the crucial role of nurses in bridging the relationship between patients and physicians and ensuring continuity of care from the hospital to the community. Nurses hold a unique position due to their direct and continuous interaction with patients; they can promptly and accurately identify clinically important issues by communicating effectively with patients [18,19]. Thus, they can help patients consult other healthcare professionals about the identified issues, or involve stakeholders in decision making [20,21]. This role is particularly crucial in the context of healthcare delivery in the Seoul metropolitan area; it pertains to children with disabilities and their families who must return to their hometowns, where rehabilitation infrastructure and systems are limited [22]. Thus, pediatric rehabilitation nurses must have a profound understanding of their role as counselors, and receive professional education that fosters the attitude and knowledge required to excel in this role. Additionally, there should be a system that connects them with healthcare professionals working in the pediatric rehabilitation field.

Fourth, pediatric rehabilitation nurses are expected to perform the role of a researcher, investigating relevant issues in the field, evaluating results, and implementing findings in their practice. This role is akin to the “researcher” role suggested by the ARN [8]. It is not only nurses that hold this view; other healthcare professionals who participated in this study expressed that despite the pressing need for research in pediatric rehabilitation nursing in clinical settings, it has remained limited [23,24]. In Korea, the Korean Academic Society of Rehabilitation Nursing, founded in 1997, has been dedicated to defining the roles of rehabilitation nurses and providing professional education [25]. However, conducting research involving children in Korea presents several challenges, as children with disabilities and their families are often categorized as vulnerable populations. Nonetheless, one study [26] found that nurses consider “participating in and conducting research” the most anticipated role of rehabilitation nurses. Our study reaffirms the significance of nurses functioning as researchers in the field of rehabilitation nursing, emphasizing their pivotal role in advancing clinical and academic aspects of the field. Establishing a support system to enhance research competencies and creating an environment conducive to research is essential for nurses to conduct meaningful research. This calls for increased support and collaboration from healthcare institutions and academic entities.

Fifth, pediatric rehabilitation nurses are expected to excel in the role of an educator, both for clients and fellow nurses. This finding aligns with the “professional practice, education, and evaluation” and “health teaching and promotion” roles proposed by the ARN [8]. Moreover, the most common response of Korean nurses was “educator” in a survey about the primary roles of rehabilitation nurses in Korea. Our finding is also consistent with previous findings that nurses are responsible for educating patients and their families, who encounter various challenges while navigating through the rehabilitation process [26,27]. However, nurses who participated in this study identified a significant barrier to fulfilling the role of an educator in clinical practice: the absence of manuals or educational programs specifically tailored to pediatric rehabilitation. This finding corresponds with that of a previous report [28]. The report found that although 88.8% of facilities claim to possess guidelines for training new graduate nurses, only 23.1–26.5% of hospitals and long-term care facilities, particularly those with fewer than 300 beds and fewer than 300 nurses, admit that they lack such guidelines for training new graduate nurses. Therefore, establishing an education support system for pediatric rehabilitation nurses emerges as a critical priority, particularly if healthcare institutions intend to effectively educate new graduate nurses, experienced nurses, healthcare professionals, and families. Additionally, given the potential variation in educational content, colleges and clinical facilities must collaborate to develop an efficient education system, explore strategies for its implementation, and support nurses effectively.

## 5. Limitation

This study has some limitations also. The study population was limited to healthcare professionals working in specific hospitals, which means that the findings may not fully capture the scope of practice across different healthcare facilities. While it is acknowledged that some roles discussed in this study, such as caregiver, team member, counselor, researcher, and educator, may overlap with the broader responsibilities of pediatric nurses, it is essential to highlight the distinctive context of pediatric rehabilitation nursing in the Korean healthcare system. Our study specifically delves into the roles expected of nurses working in pediatric rehabilitation units, a specialized area within pediatric nursing that demands a nuanced skill set and a unique set of challenges. In the Korean healthcare landscape, where nursing professionals may transition through various roles within the clinical setting, including pediatric rehabilitation, understanding the specific roles that pediatric rehabilitation nurses are expected to perform becomes crucial. Given the fluid nature of nursing responsibilities in our healthcare system, our study provides a systematic exploration of the perceptions and expectations regarding the roles of nurses within the pediatric rehabilitation context. Furthermore, our focus on pediatric rehabilitation nursing is driven by the need to inform strategies for enhancing clinical practice and shaping educational curricula. Establishing a clear understanding of the roles of pediatric rehabilitation nurses is the foundational step toward formulating effective policies, educational programs, and professional development initiatives tailored to the unique demands of pediatric rehabilitation nursing in Korea. In conclusion, our study serves as a starting point for a more in-depth exploration of the multifaceted roles of pediatric rehabilitation nurses, considering the distinct requirements and challenges posed by the Korean healthcare system and its dynamic nursing workforce. Therefore, future studies should corroborate the perspectives of pediatric rehabilitation nurses working in different healthcare settings. Based on our findings, we propose the following recommendations. First, there is a need for additional education and increased awareness about the roles and responsibilities of pediatric rehabilitation nurses. Second, to boost their skills, educational programs should be developed and offered to pediatric rehabilitation nurses possessing the required competency. Third, it is essential to implement a system that cultivates the skills of professional pediatric rehabilitation. In addition, a limitation of this study is that the pediatric rehabilitation professionals who participated in this study were all women, and it is thought that interviews will be needed from the perspective of male nurses in the future. There is also a need to confirm the role of nurses in pediatric rehabilitation units as perceived by parents of children with disabilities.

## 6. Conclusions

This study aimed to systematically explore and understand the roles that nurses working on pediatric rehabilitation units are expected to perform in Korea. Through a content analysis drawing on the experiences of healthcare professionals specializing in pediatric rehabilitation, we identified key roles, including caregivers, team members, counselors, researchers, and educators. These roles were found to be interconnected, shedding light on the dynamic and multifaceted nature of pediatric rehabilitation nursing. While the ARN [8] underscores the crucial role and responsibility of pediatric rehabilitation nurses in actively advocating for children with disabilities and their families, this role was mentioned less frequently by the participants in our study. This study is significant because it provides profound insights into the roles of pediatric rehabilitation nurses in Korea. These insights can serve as foundational data for formulating policies for healthcare personnel in pediatric rehabilitation, and provide evidence for establishing a much-needed system for certified rehabilitation nurses in Korea.

## Figures and Tables

**Table 1:** General characteristics (*n* = 12).

Participants	Age (Year)	Gender	Occupation	Total Clinical Experience (Year)	Rehabilitation Word Experience (Year)
1	41	Female	Nurse	17	12
2	51	Female	Nurse	27	15
3	35	Female	Nurse	13	10
4	35	Female	Nurse	13	10
5	38	Female	Nurse	14	10
6	35	Female	Nurse	13	10
7	34	Female	Nurse	12	11
8	34	Female	Nurse	12	12
9	35	Female	Physical therapist	13	13
10	35	Female	Occupational therapist	13	13
11	36	Female	Physiatrist	11	11
12	37	Female	Physiatrist	12	12

## Data Availability

The data used for the current study are available from the corresponding author upon reasonable request.
